# Effect of intraoperative active exploration of parathyroid glands to reduce the incidence of postoperative hypoparathyroidism, and risk factors of hypoparathyroidism after total thyroidectomy: a single-center study

**DOI:** 10.3389/fsurg.2023.1203595

**Published:** 2023-07-21

**Authors:** Bin Zhou, Feng Cheng, Xi Zhu, Lei Zhu, ZhouTing Li

**Affiliations:** Department of Thyroid Surgery, The Fifth Affiliated Hospital of Wenzhou Medical University/Lishui Central Hospital, Lishui, China

**Keywords:** total thyroidectomy, hypoparathyroidism, neck surgery, parathyroid gland, thyrothymic ligament

## Abstract

**Background:**

The risk factors for hypoparathyroidism after thyroid surgery have not been fully identified. This study analyzes the risk factors of hypoparathyroidism before and after total thyroidectomy.

**Methods:**

We retrospectively collected the clinical data of 289 patients who underwent total thyroidectomy at the Thyroid Surgery Center of Lishui Central Hospital from June 2018 to June 2020. For the anatomy and protection of parathyroid glands during the operation, one group of patients used the parathyroid avoidance method, and the other group used the active exploration method. Various risk factors affecting parathyroid dysfunction were studied using logistic regression models.

**Results:**

A total of 289 patients were included in this study. The average age of patients was 47.21 ± 11.78 years, including 57 males (19.7%) and 232 females (80.3%). There were 149 (51.6%) patients with transient hypoparathyroidism and 21 (7.3%) with permanent hypoparathyroidism. The main risk factors of hypoparathyroidism were parathyroid avoidance method (*P *= 0.005), parathyroid autotransplantation (*P *= 0.011), bilateral central neck lymph node dissection (CND) (*P *= 0.001), lymphatic metastasis (*P *= 0.039), and parathyroid in the specimen (*P *= 0.029). The main risk factors associated with permanent hypoparathyroidism were bilateral CND (*P *= 0.038), lymphatic metastasis (*P *= 0.047), parathyroid hormone (PTH) < 1.2 pg/ml within three days after surgery (*P *= 0.006).

**Conclusion:**

Hypoparathyroidism is common but mostly transient after bilateral total thyroidectomy. Compared with parathyroid avoidance method, the active exploration method during operation may reduce the incidence of postoperative hypoparathyroidism. PTH <1.2 pg/ml within three days after surgery was predictive in patients with permanent hypoparathyroidism.

## Introduction

Complete thyroidectomy is the main surgical treatment for thyroid tumors, which is mostly complicated by postoperative hypoparathyroidism (1, 2). The reported incidence of postoperative hypoparathyroidism ranges from 1.6% to over 50% (3–9), and many patients with postoperative hypoparathyroidism recover within a few months. However, there are also many patients with permanent hypoparathyroidism, with an incidence rate of 0.4%–33%. Permanent hypoparathyroidism seriously affects the quality of life of patients who require long-term calcium and vitamin D supplementation and regular monitoring of biochemical indicators. Occult hypocalcemia can even cause epileptic seizures and laryngeal spasms (10). In addition, some patients develop complications, such as basal ganglia calcification (11, 12) and cataract formation (13), and their clinical symptoms are quite painful. Therefore, measures to prevent hypoparathyroidism are of major interest to thyroid surgeons. Hypoparathyroidism is mainly due to insufficient blood supply to or accidental removal of the parathyroid (1). This study investigated the incidence and influencing factors of hypoparathyroidism after thyroid surgery in our hospital.

## Materials and methods

### Study design

Medical records of 289 patients who underwent total thyroidectomy were analyzed retrospectively. All cases were operated on in the Department of Thyroid Surgery, Lishui Municipal Central Hospital, from June 2018 to June 2020. Before surgery, all patients completed examinations, such as electrolyte, thyroid function, blood routine, liver, and kidney function, parathyroid hormone (PTH), electrocardiogram, chest x-ray, neck ultrasound, and neck computed tomography scan. In addition, all postoperative patients were routinely administered a single dose of preventive calcium supplements (calcium gluconate 2 g, intravenous injection). Non-corrected serum calcium and PTH levels were measured on days one and three after surgery. Patients were administered 1.5–3 g/day calcium carbonate and 0.5–1.5 mg/day calcitriol orally if their serum PTH and calcium levels were lower than the normal ranges. In addition, emergency intravenous infusion was added to the calcium supplementation in case of sudden onset of symptoms of hypocalcemia. Thyroid function, serum calcium, and PTH examination were repeated at two weeks, three months, and six months after surgery. The PTH detection kit of our center was produced by Roche Diagnostics GmbH, which uses electrochemiluminescence as the diagnostic method. The normal range of determination is 15–65 pg/ml (range: 1.2–5,000). Referring to the European guidelines and the standards of the American Thyroid Association (14, 15), as well as relevant literature review conclusions (16, 17), we defined postoperative hypoparathyroidism as PTH lower than normal (15 pg/ml) within 24 h after surgery. Permanent hypoparathyroidism was defined as PTH levels lower than normal for more than six months after the operation and needing drug treatment. Meanwhile, transient hypoparathyroidism was defined as stopping medication for hypoparathyroidism within six months with normal PTH and blood calcium levels.

The characteristics of patients were analyzed, including age, sex, Hashimoto thyroiditis, methods of preserving parathyroid gland in operation, malignant tumor, tumor size, lymph node metastasis, lymph node dissection, parathyroid transplantation, and pathological examination. Blood calcium and PTH were examined in all patients before, as well as day one, day three, two weeks, three months, and six months after the operation. According to the laboratory test results and symptoms of low calcium, patients with a deficiency were given calcium and vitamin D supplements. The Lishui Central Municipal Hospital Ethics Committee waived the need for written informed consent because the study was retrospective, and participants’ characteristics were anonymous.

### Surgical methods

Preventive or therapeutic neck central zone lymph node dissection was performed in all patients with thyroid cancer. The main strategy dissection included routine ipsilateral central lymph node dissection. If patients had bilateral carcinoma or combined lateral cervical lymph node metastasis, bilateral central regional lymph node dissection was performed. Our operation followed the relevant consensus in China to preserve blood supply of the parathyroid gland using the refined anatomy method and the general principle of “1 + X + 1” (at least one parathyroid gland can be strategically transplanted by identifying and reserving one parathyroid gland *in situ*) (18). During the operation, parathyroid glands that cannot be preserved *in situ* or have no blood supply after preservation undergo intramuscular autotransplantation (parathyroid tissue is cut into fine particles and planted in muscles, usually sternocleidomastoid muscles). Our center usually has two methods for anatomy and protecting the parathyroid gland during operation. Usually, the surgeon in charge will determine the surgical method on the day of the surgery, and the recording physician will describe the surgical procedure in the surgical record. By searching the medical record system and reviewing the surgical records of patients, we divide patients into two groups according to different methods. The parathyroid avoidance method involves preserving any suspicious parathyroid gland when dissecting the thyroid capsule. Meanwhile, the active exploration method entails finding the parathyroid gland and protecting its blood supply before removing the thyroid gland. Both methods conform to the principle of refined analytical methods. It should be noted that the active exploration method does not guarantee the detection of all parathyroid glands. Regardless of the method used, there is no assurance that all parathyroid glands will be found. The main difference between the two surgical methods is the timing of protecting the parathyroid glands. One approach involves protecting the parathyroid gland before removing the thyroid gland, while the other entails safeguarding the parathyroid glands during thyroid removal.

### Statistical analysis

SPSS 22.0 was used for data analysis. The Shapiro-Wilk test was used to test the normality of measurement data. Normally distributed data was expressed as mean ± standard deviation, and the difference between groups was assessed using the independent sample t-test. Meanwhile, non-normally distributed data was summarized using the median and interquartile range [M (IQR)], and the Mann-Whitney test was used to assess group differences. Group differences between categorical data were evaluated using the Chi-square test or Fisher's exact test. The Fisher's exact test was used when expected numbers were less than 1 (expected frequencies). Binary unconditional logistic regression analysis was used to identify factors influencing the occurrence of hypoparathyroidism. All tests were two-sided, and *P*-values less than 0.05 were considered statistically significant.

## Results

Patient characteristics and laboratory results are shown in Table 1. In total, 289 patients who underwent total thyroidectomy were included. The average age was 47.21 ± 11.78 years and most were female (232/289, 80.3%) and had malignant tumors (279/289, 96.54%). A total of 199 (68.9%) and 90 (31.1%) patients underwent the parathyroid avoidance method and active exploration method, respectively. Forty-two (14.5%) patients underwent parathyroid autotransplantation, 32 (76.2%) of whom experienced transient hypoparathyroidism, and 3 (7.1%) patients experienced permanent hypoparathyroidism. The two groups of patients were comparable in terms of age, gender, body mass index, tumor size, parathyroid gland transplantation rate, surgical range, etc. (*P* > 0.05). The overall incidence of transient and permanent hypoparathyroidism were 51.6% (149/289) and 7.3% (21/289), respectively. The number of patients with PTH less than the minimum detection range (1.2 pg/ml) within three days after surgery was 60 (20.8%), 13 (21.7%) of whom developed permanent hypoparathyroidism; only 8 (7.3%) of the remaining 110 patients with hypoparathyroidism developed permanent hypoparathyroidism.

**Table 1:** Patient characteristics and treatment history.

Indicators	Classification	Number of cases/composition ratio (%)
Total		289
Hypoparathyroidism	No	119 (41.2)
	Yes	170 (58.8)
Permanent hypoparathyroidism	No	268 (92.7)
	Yes	21 (7.3)
Gender	Male	57 (19.7)
	Female	232 (80.3)
Age, years		16–85 (47.21 ± 11.78)
BMI		16.16–37.77 (24.44 ± 3.54)
Character	Benign	10 (3.46)
	Malignant	279 (96.54)
Hashimoto's thyroiditis	No	167 (57.8)
	Yes	122 (42.2)
Anatomy and protection of parathyroid glands methods	avoidance method	199 (68.9)
	active exploration method	90 (31.1)
Parathyroid autotransplantation	No	247 (85.5)
	Yes	42 (14.5)
Range of the lymph node dissection	no CND/ipsilateral CND	83 (28.7)
	bilateral CND	206 (71.3)
PTH whin three days after surgery,(pg/ml)	<1.2	60 (20.8)
	1.2–15	110 (38.1)
	>15	119 (41.2)
The maximum diameter of the tumor, (cm)		2–60 (13.13 ± 9.94)
Tumor site	Unilateral	127 (45.2)
	Bilateral	154 (54.8)
Mulifocality	Single	46 (16.4)
	Multifocal	235 (83.6)
Lymphatic metastasis	No	115 (40.9)
	Yes	166 (59.1)
Parathyroid in the specimen	No	219 (75.8)
	Yes	70 (24.2)

CND, central neck lymph node dissection.

Univariate analysis (Table 2) showed that postoperative hypoparathyroidism was more common in Hashimoto's thyroiditis (*P *= 0.046), parathyroid avoidance method (*P *= 0.010), parathyroid autotransplantation (*P *= 0. 013), bilateral central neck lymph node dissection (CND) (*P *= 0.001), multifocality (*P *= 0.037), lymphatic metastasis (*P *= 0.039), and parathyroid in the specimen (*P *= 0.006). There was no significant correlation between sex, age, body mass index (BMI), tumor diameter, and tumor location. The multivariate analysis (Table 3) showed that postoperative hypoparathyroidism was more common in the parathyroid avoidance method (*P *= 0.005), parathyroid autotransplantation (*P *= 0.011), bilateral CND (*P *= 0.001), lymphatic metastasis (*P *= 0.039), and parathyroid in the specimen (*P *= 0.029).

**Table 2:** Results of univariate analysis of postoperative hypoparathyroidism.

Indicators	None (*n* = 119)(%)	Yes (*n* = 170)(%)	t/2χ	*P*
Gender			2.759	0.097
Male	29 (50.9)	28 (49.1)		
Female	90 (38.8)	142 (61.2)		
Age, years	47.37 ± 12.40	47.11 ± 11.37	0.187	0.852
BMI	24.15 ± 3.70	24.65 ± 3.42	−1.170	0.243
Hashimoto's thyroiditis			3.972	**0** **.** **046**
YES	42 (34.4)	80 (65.6)		** **
NO	77 (46.1)	90 (53.9)		** **
Anatomy and protection of parathyroid glands methods			6.584	**0**.**010**
Avoidance method	72 (36.2)	127 (63.8)		** **
Active exploration method	47 (52.2)	43 (47.8)		** **
Parathyroid autotransplantation			6.119	**0**.**013**
YES	10 (23.8)	32 (76.2)		** **
NO	109 (44.1)	138 (55.9)		** **
Range of the lymph node dissection			11.475	**0**.**001**
No CND/ipsilateral CND	47 (56.6)	36 (43.4)		** **
bilateral CND	72 (34.9)	134 (65.1)		** **
The maximum diameter of the tumorr,(cm)	12.08 ± 9.10	13.85 ± 10.44	−1.463	0.145
Tumor site			3.373	0.066
Unilateral	44 (34.6)	83 (65.4)		** **
Bilateral	70 (45.5)	84 (54.5)		** **
Mulifocality			4.331	**0**.**037**
Single	25 (54.3)	21 (45.7)		** **
Multifocal	89 (37.9)	146 (62.1)		** **
Lymphatic metastasis			4.252	**0**.**039**
YES	59 (35.6)	107 (64.4)		** **
NO	55 (47.8)	60 (52.2)		** **
Parathyroid in the specimen			7.511	**0**.**006**
YES	19 (27.1)	51 (72.9)		** **
NO	100 (45.7)	119 (54.3)		** **

Bold indicates the significant *P*-value <0.05.

**Table 3:** Multivariate regression analysis of postoperative hypoparathyroidism.

Indicators	B	SE	Wald	*P*	OR	95% CI
Anatomy and protection of parathyroid glands methods	0.796	0.282	7.956	**0**.**005**	2.216	1.275–3.851
Range of the lymph node dissection	0.941	0.284	10.983	**0**.**001**	2.563	1.469–4.472
Parathyroid autotransplantation	1.044	0.411	6.446	**0**.**011**	2.840	1.269–6.355
Lymphatic metastasis	0.543	0.262	4.280	**0**.**039**	1.721	1.029–2.877
Parathyroid in the specimen	0.698	0.319	4.782	**0**.**029**	2.009	1.075–3.755

Bold indicates the significant *P*-value <0.05.

Univariate analysis was conducted in patients with or without permanent hypoparathyroidism (Table 4). The results showed that permanenthypoparathyroidism was more likely to occur under the following conditions: bilateral CND (*P *= 0.012), lymphatic metastasis (*P *= 0.048), and parathyroid in the specimen (*P *= 0.038). The multivariate analysis (Table 5) showed that permanent hypoparathyroidism is more likely to occur in bilateral CND (*P *= 0.038) and lymphatic metastasis (*P *= 0.047).

**Table 4:** Univariate analysis of permanent hypoparathyroidism.

Indicators	None (*n* = 268)	Yes (*n* = 21)	t/χ^2^	*P*
Gender			0.239	0.625
Male	52 (91.2)	5 (8.8)		
Female	216 (93.1)	16 (6.9)		
Age, years	47.08 ± 11.81	48.95 ± 11.53	−0.701	0.484
BMI	24.41 ± 3.59	24.85 ± 2.82	−0.547	0.585
Hashimoto's thyroiditis			0.271	0.603
Yes	112 (91.8)	10 (8.2)		
No	156 (93.4)	11 (6.6)		
Anatomy and protection of parathyroid glands methods			1.545	0.214
Avoidance method	182 (92.4)	17 (7.6)		
Active exploration method	86 (95.6)	4 (4.4)		
Parathyroid autotransplantation			0.001	0.973
Yes	39 (92.9)	3 (7.1)		
No	229 (92.7)	18 (7.3)		
Range of the lymph node dissection			6.349	**0** **.** **012**
No CND/ipsilateral CND	82 (98.8)	1 (1.2)		
bilateral CND	186 (90.3)	20 (9.7)		
The maximum diameter of the tumor,(cm)	13.09 ± 10.09	13.68 ± 7.82	−0.250	0.803
Tumor site			0.201	0.654
Unilateral	117 (92.1)	10 (7.9)		
Bilateral	144 (93.5)	10 (6.5)		
Mulifocality			0.030	0.864
Single	43 (93.5)	3 (6.5)		
Multifocal	218 (92.8)	17 (7.2)		** **
Lymphatic metastasis			3.900	**0**.**048**
Yes	150 (90.4)	16 (9.6)		** **
No	111 (96.5)	4 (3.5)		** **
Parathyroid in the specimen			4.285	**0**.**038**
Yes	61 (87.1)	9 (12.9)		
No	207 (94.5)	12 (5.5)		

Bold indicates the significant *P*-value <0.05.

**Table 5:** Multivariate regression analysis of permanent hypoparathyroidism.

Indicators	B	SE	Wald	*P*	OR	95% CI
Range of the lymph node dissection	2.150	1.037	4.296	**0**.**038**	8.582	1.124∼65.520
Lymphatic metastasis	1.148	0.577	3.956	**0**.**047**	3.151	1.017∼9.764

Bold indicates the significant *P*-value <0.05.

When comparing patients with transient and permanent hypoparathyroidism, we added the variable PTH within three days after surgery <1.2 pg/ml.

Univariate analysis (Table 6) showed that patients with bilateral CND (*P *= 0.049) and PTH within three days after surgery <1.2 pg/ml (*P *= 0.006) were more likely to develop permanent hypoparathyroidism. Meanwhile, the multivariate analysis (Table 7) showed that only the latter factor significantly affected permanent hypoparathyroidism (*P *= 0.006).

**Table 6:** Univariate analysis of transient and permanent hypoparathyroidism.

Indicators	None (*n* = 149)(%)	Yes (*n* = 21)(%)	t/χ^2^	*P*
Gender			0.938	0.333
Male	23 (82.2)	5 (17.8)		
Female	126 (88.7)	16 (11.3)		
Age, years	46.85 ± 11.36	48.95 ± 11.53	−0.794	0.428
BMI	24.62 ± 3.50	24.85 ± 2.82	−0.291	0.771
Hashimoto's thyroiditis			0.003	0.956
Yes	70 (87.5)	10 (12.5)		
No	79 (87.8)	11 (12.2)		
Anatomy and protection of parathyroid glands methods			0.495	0.482
Avoidance method	110 (86.6)	17 (13.4)		
Active exploration method	39 (90.7)	4 (9.3)		
Parathyroid autotransplantation			0.323	0.570
Yes	29 (90.6)	3 (9.4)		
No	120 (87.0)	18 (13.0)		
Range of the lymph node dissection			3.868	**0** **.** **049**
No CND/ipsilateral CND	35 (97.2)	1 (2.8)		
Bilateral CND	114 (85.1)	20 (14.9)		
The maximum diameter of the tumor,(cm)	13.87 ± 10.75	13.68 ± 7.82	−0.073	0.942
Tumor site			0.001	0.977
Unilateral	73 (88.0)	10 (12.0)		
Bilateral	74 (88.1)	10 (11.9)		
Mulifocality			0.122	0.727
Single	18 (85.7)	3 (14.3)		
Multifocal	129 (88.4)	17 (11.6)		** **
Lymphatic metastasis			2.504	0.114
Yes	91 (85.0)	16 (15.0)		** **
No	56 (93.3)	4 (6.7)		** **
Parathyroid in the specimen			1.886	0.170
Yes	42 (82.4)	9 (17.6)		
No	107 (89.9)	12 (10.1)		
PTH whin three days after surgery			7.429	**0**.**006**
1.2–15(pg/ml)	102 (85.0)	8 (15.0)		
<1.2(pg/ml)	47(78.3)	13(21.7)		

Bold indicates the significant *P*-value <0.05.

**Table 7:** Multivariate regression analysis of transient and permanent hypoparathyroidism.

	B	SE	Wald	*P*	OR	95% CI
PTH whin three days after surgery <1.2(pg/ml)	1.337	0.491	7.419	**0**.**006**	3.806	1.455∼9.959
Range of the lymph node dissection	1.937	1.052	3.388	0.066	6.935	0.882∼54.536

Bold indicates the significant *P*-value <0.05.

## Discussion

Hypoparathyroidism is one of the most common surgical complications of total thyroidectomy, which mostly results from a cut in the blood supply of the parathyroid gland during the operation. After six months of follow-up, transient and persistent hypoparathyroidism in this study accounted for 51.6% and 7.3%, respectively. The multivariate logistic regression analysis conducted on 289 patients undergoing total thyroidectomy in our center showed the parathyroid avoidance method, parathyroid autotransplantation, bilateral CND, lymphatic metastasis, and parathyroid in specimens were associated with postoperative hypoparathyroidism. In addition, bilateral CND and lymphatic metastasis were associated with permanent hypoparathyroidism. For patients with postoperative hypoparathyroidism, PTH within three days after surgery cannot be measured had predictive significance for the occurrence of permanent hypoparathyroidism.

Preserving the parathyroid gland during thyroidectomy is a major concern for thyroid surgeons because even benign diseases cannot guarantee normal parathyroid gland function after thyroidectomy. If the tumor is large, invasive, or has extensive lymph node metastasis, it is challenging to maintain the integrity of the parathyroid glands. According to a systematic review, the median incidence of temporary and permanent hypoparathyroidism were 27% and 1%, and the highest rates were 38% and 3%, respectively (19). The incidence of transient hypoparathyroidism in this study was relatively high, partly because all the included patients underwent bilateral total thyroidectomy. Additionally, the proportion of patients with thyroid cancer in this study was as high (96.5%). We performed routine preventive central lymph node dissection on patients with thyroid cancer, which also increased the incidence of hypoparathyroidism.

This study found that the incidence of postoperative hypoparathyroidism varied by the parathyroid anatomy and protection methods. The rate of transient hypoparathyroidism caused by the active exploration method was significantly lower than that caused by the avoidance method, which may be due to the following principal reasons. The active exploration method can better preserve the blood supply of the parathyroid gland. For example, the blood supply of the superior parathyroid gland mostly comes from the terminal branch of the inferior thyroid artery or the posterior branch of the superior thyroid artery (20). When using the parathyroid avoidance method, these branches are easily ligated first, resulting in damage to the blood supply of the superior parathyroid gland. For some of the lower polar parathyroid glands, the blood supply comes from the thyrothymic ligament (21).The active exploration method can better preserve the blood supply between the thyrothymic ligament and the lower polar parathyroid glands. Based on this result, ensuring that the blood supply of the parathyroid gland is not interrupted is important for preserving the parathyroid gland. Therefore, one should use the active exploration method during the operation to preserve thebranchs of the thyroid artery and thyrothymic ligament. We found that the incidence of permanent hypoparathyroidism caused by the active exploration method (4.4%) was also lower than the parathyroid avoidance method (8.5%), but the difference was not statistically significant (*P* = 0.21), which may be related to the low number of patients with permanent hypoparathyroidism.

Lymph node dissection in the VI area of the neck can easily destroy the supply of blood vessels of the parathyroid gland, leading to hypoparathyroidism to a certain extent (22, 23). Existing articles have different views on how to perform cervical lymph node dissection. Some people suggest that preventive dissection will not significantly reduce the recurrence and metastasis rate of the tumor but will increase the risk of parathyroid damage (24). This case is similar to other studies, suggesting that lymph node dissection is a risk factor for temporary and persistent hypoparathyroidism. Therefore, the operation procedure should be strictly observed and conducted to reduce injury to the parathyroid glands during the operation. Where possible, other chromogenic substances, such as Nano-C, can be used to stain the parathyroid gland (25, 26). In recent years, fluorescence imaging—relying both on parathyroid gland autofluorescence under near-infrared light—and angiography using the fluorescent dye indocyanine green-has been used to reduce the risk of iatrogenic parathyroid injury during thyroid and parathyroid resections. Parathyroid autofluorescence was considered better than indocyanine green angiography for localizing parathyroid glands, whereas indocyanine green angiography was deemed superior in assessing parathyroid perfusion (27–29). But there are no published guidelines exist regarding its use.Some surgeons prefer to remove thyroid cancer and perform central chamber lymph node dissection, but this increases the risk of damage to complex vascular connections in parathyroid glands.

This study showed that patients with lymph node metastasis have a high incidence of transient and permanent hypoparathyroidism after surgery, consistent with the disease characteristics of thyroid cancer. Firstly, in patients with lymph node metastasis, the scope of lymph node dissection usually includes the bilateral VI and even the lateral neck area. Secondly, in patients with lymph node metastasis in the central region, it is more likely to damage the blood supply of the parathyroid gland during the resection because they are usually accompanied by abnormally swollen lymph nodes.

Our study also showed that autoparathyroid transplantation has some effect on the occurrence of postoperative hypoparathyroidism, but it has no significant correlation with permanent hypoparathyroidism, indicating that the transplanted parathyroid gland needs a certain time to survive and function. Some studies also confirmed that autologous parathyroid transplantation can prevent permanent hypoparathyroidism (3, 30). In addition, some researchers recommend conventional autotransplantation of parathyroid glands (31). Furthermore, we observed that accidental parathyroidectomy is a significant risk factor for transient hypoparathyroidism but was not associated with persistent hypoparathyroidism. A complete parathyroid gland is widely believed to maintain the body's blood calcium (32). Lee et al. surveyed 2,636 patients and found that inadvertent parathyroidectomy did not affect postoperative hypoparathyroidism after surgery (33). However, even if a single reserved parathyroid gland seems feasible, it may also lose its function, resulting in postoperative hypoparathyroidism (34). Therefore, the parathyroid gland should be retained as much as possible in surgical practice, and the parathyroid gland should be carefully searched in the anatomical specimen. If the parathyroid gland is found to be resected by mistake, timely parathyroid gland autotransplantation should be performed.

This study shows that patients with lower PTH levels (less than 1.2 pg/ml) within three days after surgery had a higher risk of permanent hypoparathyroidism, similar to previous studies (35). For patients with PTH still below detectable levels on the third day, it is necessary to strengthen calcium and vitamin D3 supplementation. A lack of timely calcium supplementation or follow-up may cause patients to lose the opportunity to recover their parathyroid function.

## Summary

Hypoparathyroidism commonly occurs after total thyroidectomy and is mostly temporary. Parathyroid autotransplantation, bilateral CND, lymphatic metastasis, and parathyroid in the specimen are objective risk factors for postoperative hypoparathyroidism.

Understanding the complex vascular structure around the parathyroid gland can prevent hypoparathyroidism after thyroidectomy. Compared with parathriod avoidance method, the active exploration method may reduce the incidence of postoperative hypoparathyroidism. Few cases of transient hypoparathyroidism evolve into permanent hypoparathyroidism, bilateral CND, lymphatic metastasis, and PTH within three days after surgery <1.2 pg/ml were the risk factors for postoperative permanent hypoparathyroidism. More attention needs to be paid to these patients, and timely calcium and vitamin D supplementation after surgery can improve their symptoms.More importantly, we need to make sufficient efforts to reduce the occurrence of this situation.

The limitations of this study are that the number of patients with permanent hypoparathyroidism was relatively small, and a few patients recovered their parathyroid function after a long time (over six months and even one year), which may bias the result of the study. Our study is also limited by some factors inherent to retrospective research, including the inability to specify and standardize subject recruitment and data collection in advance.

## Data Availability

The original contributions presented in the study are included in the article/Supplementary Material, further inquiries can be directed to the corresponding author.
